# Selective Confidence-Guided Projection-Based Encoding for Medical Image Classification

**DOI:** 10.3390/jimaging12090436

**Published:** 2026-09-11

**Authors:** Tao Chen, Chuan Zhou, Yifan Wang, Lubomir M. Hadjiiski, Qian Dong

**Affiliations:** Department of Radiology, University of Michigan, Ann Arbor, MI 48109, USA

**Keywords:** medical image classification, knowledge distillation, reliability-aware supervision, gradient conflict, structured priors

## Abstract

Deep neural networks have achieved strong performance in medical image classification, but their deployment may be constrained by the computational cost of high-capacity models. Knowledge distillation (KD) addresses this problem by transferring knowledge from a teacher to a lightweight student. However, the reliability of teacher supervision may vary across samples, potentially introducing noisy guidance and local conflicts with ground-truth supervision. We propose Selective Confidence-guided Projection-based Encoding (SCOPE), a conflict-aware KD framework comprising Selective Relation Alignment (SRA) and Gradient Conflict Resolution (GCR). SRA constructs reliability-aware relational supervision by combining teacher-derived relations with dataset-specific auxiliary priors, whereas GCR removes distillation-gradient components that conflict with the classification objective. Experiments on nine medical image datasets and multiple teacher–student architectures demonstrate competitive predictive performance, improved training stability, and low computational overhead.

## 1. Introduction

Deep neural networks have achieved promising performance in medical image classification [1,2], but their deployment is often constrained by the computational and memory requirements of high-capacity models [3]. These limitations are particularly relevant in clinical environments, where available hardware and dataset characteristics may vary across institutions. Lightweight models that maintain reliable performance across heterogeneous medical imaging tasks are therefore needed.

Knowledge distillation (KD) transfers knowledge from a high-capacity teacher to a compact student [4,5,6,7]. Existing methods transfer intermediate features, inter-sample relations, or softened predictions [8,9,10,11,12,13]. KD has also been extended to biomedical applications through medical priors and task-specific constraints [14,15,16,17,18]. Nevertheless, conventional KD commonly assumes that teacher predictions are reliable. This assumption may not hold for previously unseen samples, where erroneous teacher predictions can introduce noisy supervision. Nevertheless, entirely removing teacher guidance may also result in the loss of valuable relational and semantic information. The central challenge is therefore to retain reliable teacher knowledge while suppressing misleading guidance.

Previous studies have addressed unreliable supervision through loss perturbation [19], pseudo-label denoising [20], supervision refinement [21,22], uncertainty-aware distillation [23,24] or confidence-aware learning strategies [25]. Confidence-aware methods generally adjust the strength of teacher supervision at the sample or loss level, whereas relation-based distillation transfers the structural relationships among samples. However, these approaches do not explicitly construct pairwise relational targets by jointly considering teacher reliability. Gradient-conflict methods such as PCGrad, GradDrop, and CAGrad [26,27,28] have been developed primarily for multi-task optimization. Their direct application does not specifically prioritize the ground-truth classification objective over potentially unreliable distillation supervision. Therefore, an unresolved question is how to jointly control the reliability of relational knowledge and prevent conflicting distillation gradients from degrading the supervised classification objective.

**Figure 1:**
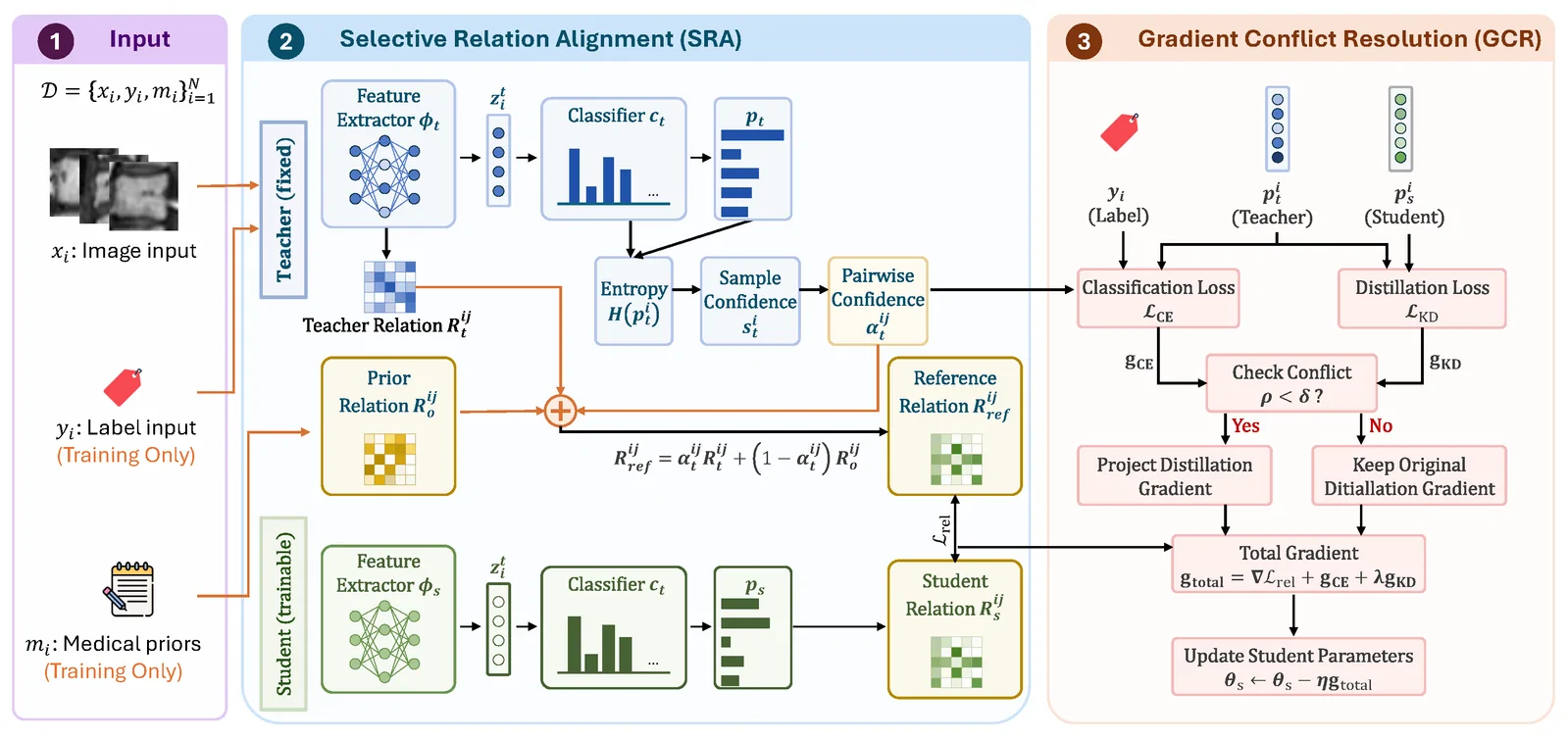
Overview of the proposed SCOPE framework. Given a labeled dataset $D={\left\{\left(x_{i},y_{i}\right)\right\}}_{i=1}^{N}$, the teacher and student networks extract feature representations and generate class predictions. SRA constructs reliability-aware relational supervision by combining the teacher-derived relation $R_{ij}^{t}$ with an auxiliary relation $R_{ij}^{o}$ according to the pairwise confidence $\alpha_{ij}^{t}$. The auxiliary relation is constructed from medical prior information when available; otherwise, it is derived from a normalized Euclidean distance between preprocessed input images. The student relation $R_{ij}^{s}$ is aligned with the resulting reference relation $R_{ij}^{\mathrm{ref}}$. GCR detects local inconsistencies between the classification and distillation gradients and conditionally corrects the distillation gradient before updating the student parameters.

To address this problem, we formulate SCOPE (Selective COnfidence-guided Projection-based Encoding) as a selective knowledge-transfer framework designed to preserve informative teacher knowledge while limiting the influence of less informative guidance on student learning. Teacher-derived relations provide valuable structural information for knowledge transfer, while their informativeness may vary across sample pairs. SRA therefore uses pairwise confidence to adaptively regulate the contribution of teacher-derived relations when constructing the reference relation. GCR therefore preserves the classification gradient as the primary learning direction and selectively removes only the conflicting component of the distillation gradient. When the two gradients are directionally consistent, the original distillation gradient is retained, allowing teacher knowledge to continue contributing to student optimization; when a conflict occurs, only the component opposing the classification gradient is removed. In this way, GCR retains the contribution of compatible distillation signals while preventing conflicting components from interfering with ground-truth supervised learning. SRA and GCR implement a unified selective-transfer principle that regulates both the relational information transferred from the teacher and how the resulting distillation signal influences student optimization. An overview of the proposed SCOPE framework is presented in Figure 1. The main contributions are as follows:

- We propose a confidence-aware relational distillation strategy that uses teacher confidence to weight and combine teacher relations with dataset-specific auxiliary-prior relations.
- We introduce a classification-priority gradient correction method that keeps the classification gradient unchanged while only adjusting conflicting KD gradients.
- We formulate a selective knowledge-transfer framework that adaptively regulates teacher-derived relational knowledge and constrains conflicting distillation gradients to preserve the ground-truth classification objective.

## 2. Related Work

Knowledge distillation methods differ primarily in the form of knowledge transferred from the teacher. Response-based methods use softened output distributions, whereas feature-based methods align intermediate representations or attention patterns [29,30]. Relation-based methods instead transfer structural relationships among samples or representations [31]. Although these approaches differ in what is transferred, they generally do not explicitly account for variations in the reliability of teacher supervision across sample pairs.

Uncertainty- and confidence-aware distillation methods reduce the influence of unreliable teacher outputs through confidence weighting, uncertainty-based masking, or adaptive supervision [32]. Existing methods primarily apply such regulation to individual predictions, classes, or distillation losses. In contrast, SRA uses the confidence of both samples to regulate the construction of each relational target.

Knowledge distillation has also been applied to medical imaging tasks, including image classification, physiological measurement, radiology report generation, and whole-slide image analysis [14,15,16,17]. Knowledge-enhanced medical image classification methods have incorporated explicit clinical rules or structured domain knowledge into representation learning [33]. The present study focuses on a related but distinct question: how complementary pairwise information can be incorporated into relational distillation when the reliability of teacher supervision varies across sample pairs. In SCOPE, the auxiliary relation is constructed from medical prior information when such information is available; otherwise, it is derived from a normalized Euclidean distance between preprocessed input images, providing a non-learned source of pairwise information. This design allows SRA to construct an auxiliary relation without requiring the same type of structured metadata for every dataset.

Gradient-conflict methods such as PCGrad, GradDrop, and CAGrad modify optimization directions when gradients from multiple objectives interfere with one another [26,27,28]. These methods were primarily developed for multi-task optimization and seek to reconcile multiple objectives without assigning the ground-truth classification objective a fixed priority. GCR instead preserves the classification gradient and modifies only the conflicting component of the distillation gradient. SCOPE combines this classification-priority correction with pairwise confidence-aware relational supervision.

## 3. Materials and Methods

### 3.1. Problem Formulation and Framework Overview

Let $D={\left\{\left(x_{i},y_{i}\right)\right\}}_{i=1}^{N}$ denote a labeled dataset, where $x_{i}$ is the input image and $y_{i}$ is the corresponding class label. In addition to the teacher-derived relation, SCOPE constructs an auxiliary relation $R_{ij}^{o}$ for each sample pair (i,j). The source of this relation depends on the availability of structured sample-level information. When suitable medical prior information is available, each sample is associated with a prior representation $m_{i}\in R^{d_{r}}$, from which the pairwise auxiliary relation is computed. When such prior information is unavailable, the auxiliary relation is instead derived from the normalized Euclidean distance between the corresponding preprocessed input images. The teacher and student models are denoted by $f_{t}=\left(\phi_{t},c_{t}\right)$ and $f_{s}=\left(\phi_{s},c_{s}\right)$, respectively, where ϕ represents the feature extractor and *c* represents the classifier. Their feature representations, classification logits, and class-probability distributions are defined as

(1) $$\begin{array}{cccccc} z_{t}^{i}\; & =\phi_{t}\left(x_{i}\right),\; & o_{t}^{i}\; & =c_{t}\left(z_{t}^{i}\right),\; & p_{t}^{i}\; & =\mathrm{Softmax}(o_{t}^{i}),\\ z_{s}^{i}\; & =\phi_{s}\left(x_{i}\right),\; & o_{s}^{i}\; & =c_{s}\left(z_{s}^{i}\right),\; & p_{s}^{i}\; & =\mathrm{Softmax}(o_{s}^{i}), \end{array}$$

where $z_{i}^{t}\in R^{d_{t}}$ and $z_{i}^{s}\in R^{d_{s}}$ denote the teacher and student feature representations, respectively. Here, $d_{t}$ and $d_{s}$ are architecture-dependent feature dimensions.$o_{t}^{i},o_{s}^{i}\in R^{C}$ are classification logits, and $p_{t}^{i},p_{s}^{i}\in R^{C}$ are the corresponding probability distributions over *C* classes.

As illustrated in Figure 1, SCOPE consists of two components: Selective Relation Alignment (SRA) and Gradient Conflict Resolution (GCR). SRA constructs reliability-aware relational supervision by combining teacher-derived relations with dataset-specific auxiliary priors, whereas GCR corrects distillation gradients that conflict with the ground-truth classification objective.

For a mini-batch of *B* samples, the classification loss is defined as

(2) $$L_{\mathrm{CE}}=-\frac{1}{B}\sum_{i=1}^{B}\sum_{c=1}^{C}y_{i,c}\log p_{s,c}^{i},$$

where $y_{i,c}$ denotes the one-hot label indicator and $p_{s,c}^{i}$ denotes the student probability for class *c*. The temperature-based distillation loss is defined as

(3) $$L_{\mathrm{KD}}\left(T\right)=\frac{1}{B}\sum_{i=1}^{B}\mathrm{KL}\left[\mathrm{Softmax}\left(\frac{o_{t}^{i}}{T}\right)\;∥\;\mathrm{Softmax}\left(\frac{o_{s}^{i}}{T}\right)\right],$$

where *T* denotes the distillation temperature. The training objective contains the relational alignment, classification, and distillation terms:

(4) $$L_{\mathrm{base}}=L_{\mathrm{rel}}+\beta L_{\mathrm{CE}}+\lambda \gamma T^{2}L_{\mathrm{KD}}\left(T\right).$$

where β, γ and λ are balancing coefficients.

### 3.2. Selective Relation Alignment

Because direct feature matching can be sensitive to variations in feature scale and distribution during training, SRA instead transfers inter-sample relational information. Teacher-derived relations are weighted according to prediction confidence, allowing uncertain teacher supervision to be complemented by an auxiliary relation. The auxiliary relation is constructed according to the availability of medical prior information for each dataset. When suitable medical prior information is available, it is encoded into a prior representation $m_{i}$. For metadata-based priors, continuous variables are standardized using statistics estimated from the corresponding training partition, whereas categorical variables are encoded using one-hot representations. The resulting dimensionality $d_{r}$ is determined by the number of standardized continuous variables and the dimensions of the one-hot-encoded categorical variables for the corresponding dataset. When suitable medical prior information is unavailable, no separate prior vector is constructed; instead, the auxiliary relation is computed directly from the normalized Euclidean distance between the corresponding vectorized, preprocessed input images. This image-based relation is deterministic and does not introduce an additional trainable network.

We next estimate the reliability of the teacher prediction for each sample using its predictive entropy:

(5) $$H\left(p_{t}^{i}\right)=-\sum_{c=1}^{C}p_{t,c}^{i}\log p_{t,c}^{i}.$$

Lower entropy indicates greater teacher confidence. We therefore define the sample-level confidence as $s_{t}^{i}=\exp\left[-H\left(p_{t}^{i}\right)/\log C\right]$ and the corresponding pairwise confidence as $\alpha_{t}^{ij}=s_{t}^{i}s_{t}^{j}$. Its association with teacher classification accuracy is empirically evaluated in Section 4.4. The teacher and student relations are computed using cosine similarity, whereas the auxiliary relation is constructed according to the availability of medical prior information.

(6) $$\begin{array}{cc} R_{t}^{ij}\; & =\frac{\langle z_{t}^{i},z_{t}^{j}\rangle }{{∥z_{t}^{i}∥}_{2}{∥z_{t}^{j}∥}_{2}},\;R_{s}^{ij}=\frac{\langle z_{s}^{i},z_{s}^{j}\rangle }{{∥z_{s}^{i}∥}_{2}{∥z_{s}^{j}∥}_{2}},\\ R_{ij}^{o}\; & =\left\{\begin{array}{cc} \exp\left(-\frac{d_{m}\left(m_{i},m_{j}\right)}{\tau }\right),\; & \mathrm{if}\;\mathrm{medical}\;\mathrm{prior}\;\mathrm{information}\;\mathrm{is}\;\mathrm{available},\\ \exp\left(-\frac{d_{x}\left(x_{i},x_{j}\right)}{\tau }\right),\; & \mathrm{otherwise}. \end{array}\right. \end{array}$$

where $d_{m}\left(\cdot ,\cdot \right)$ and $d_{x}\left(\cdot ,\cdot \right)$ denote the Euclidean distances between medical-prior representations and between vectorized, preprocessed input images, respectively. In both cases, the resulting pairwise distances are normalized to [0,1] within each mini-batch before being transformed into the auxiliary relation $R_{ij}^{o}$ using the exponential kernel. Thus, more similar sample pairs receive larger auxiliary-relation values. The parameter τ>0 controls the decay rate and is fixed at τ=1.0 in all experiments. The operators 〈·,·〉 and ${∥\cdot ∥}_{2}$ denote the inner product and Euclidean norm, respectively. The reliability-aware reference relation is then defined as:

(7) $$R_{\mathrm{ref}}^{ij}=\alpha_{t}^{ij}R_{t}^{ij}+\left(1-\alpha_{t}^{ij}\right)R_{o}^{ij}.$$

Accordingly, teacher-derived relations are assigned greater weight when teacher confidence is high, whereas the auxiliary prior relation exerts a stronger influence under low-confidence predictions. In the No-Prior setting, the auxiliary relation $R_{o}^{ij}$ is removed and the reference relation reduces to $R_{\mathrm{ref}}^{ij}=R_{t}^{ij}$. SRA therefore becomes teacher-based relational distillation without auxiliary prior information. The relational alignment loss is calculated over all distinct sample pairs within a mini-batch:

(8) $$L_{\mathrm{rel}}=\frac{1}{B(B-1)}\sum_{\begin{array}{c} i,j=1\\ i\neq j \end{array}}^{B}{\left(R_{s}^{ij}-R_{\mathrm{ref}}^{ij}\right)}^{2}.$$

SRA therefore preserves informative teacher-derived relations while reducing the influence of uncertain supervision.

### 3.3. Gradient Conflict Resolution

Although SRA adaptively regulates the relational knowledge transferred from the teacher, this regulation does not necessarily ensure directional agreement between the classification and distillation objectives during optimization. Their gradients may still exhibit local directional conflicts. GCR therefore preserves the classification gradient as the primary learning direction while removing only the conflicting component of the distillation gradient. Let $\theta_{s}$ denote the parameters of the student network. The gradients associated with the classification and distillation objectives are defined as follows:

(9) $$g_{\mathrm{CE}}=\beta \nabla_{\theta_{s}}L_{\mathrm{CE}},\;g_{\mathrm{KD}}=\gamma T^{2}\nabla_{\theta_{s}}L_{\mathrm{KD}}.$$

Their directional agreement is measured using cosine similarity:

(10) $$\rho =\frac{\langle g_{\mathrm{CE}},g_{\mathrm{KD}}\rangle }{∥g_{\mathrm{CE}}{∥}_{2}{∥g_{\mathrm{KD}}∥}_{2}+\epsilon },$$

where ϵ is a small constant for numerical stability. A gradient conflict is detected when ρ<δ. Although δ can be varied, we set δ=0 as the default threshold for all datasets. This choice follows directly from the geometry of the two gradients. ρ<0 indicates that the classification and distillation gradients have a negative inner product and therefore point in conflicting directions, whereas ρ≥0 indicates no directional conflict according to the cosine-similarity criterion. Accordingly, δ=0 was fixed a priori rather than selected through dataset-specific tuning. This geometrically defined threshold provides a consistent decision criterion across datasets and avoids the need for dataset-specific threshold search. The sensitivity of GCR to alternative threshold values is further examined in Section 4.3. The distillation gradient is conditionally corrected according to

(11) $${\tilde{g}}_{\mathrm{KD}}=\left\{\begin{array}{cc} g_{\mathrm{KD}}-\frac{\langle g_{\mathrm{KD}},g_{\mathrm{CE}}\rangle }{∥g_{\mathrm{CE}}{∥}_{2}^{2}+\epsilon }g_{\mathrm{CE}},\; & \mathrm{if}\;\rho <\delta ,\\ g_{\mathrm{KD}},\; & \mathrm{otherwise}. \end{array}\right.$$

when ρ<δ, the component of the distillation gradient that conflicts with the classification gradient is removed; otherwise, the original distillation gradient is retained. Thus, Equation (11) implements a hard correction once the conflict criterion is satisfied. A continuous correction strategy that adaptively scales the projection according to the degree of gradient conflict is further examined in the ablation study. The final update direction is

(12) $$g_{\mathrm{total}}=\nabla_{\theta_{s}}L_{\mathrm{rel}}+g_{\mathrm{CE}}+\lambda {\tilde{g}}_{\mathrm{KD}},$$

where λ controls the contribution of the corrected distillation gradient. The student parameters are updated according to

(13) $$\theta_{s}\leftarrow \theta_{s}-\eta g_{\mathrm{total}},$$

where η denotes the learning rate.

SRA improves the reliability of relational supervision, whereas GCR prevents conflicting components of the distillation gradient from interfering with the ground-truth classification objective.

## 4. Experiments

### 4.1. Experiment Setting

#### 4.1.1. Datasets and Experimental Design

The proposed framework was evaluated on nine medical image classification datasets, comprising eight publicly available benchmarks and one institutionally collected Multiple Myeloma dataset. These datasets comprised between three and seven classes and varied substantially in size, ranging from 488 to 25,000 images. Specifically, the datasets included LC25000 (https://www.kaggle.com/datasets/javaidahmadwani/lc25000 (accessed on 1 November 2024)) (25,000 images, five classes), HAM10000 (https://www.kaggle.com/datasets/vrindaat/ham10000-dataset (accessed on 1 November 2024)) (10,015 images, seven classes), Chaoyang (https://bupt-ai-cz.github.io/HSA-NRL/ (accessed on 1 November 2024)) (6160 images, four classes), Multiple Myeloma (1816 images, four classes), Brain Tumor (https://www.kaggle.com/datasets/masoudnickparvar/brain-tumor-mri-dataset (accessed on 1 November 2024)) (3264 images, four classes), Kidney (https://github.com/Ritesh18117/Detection-and-Classification-of-Kidney-Diseases-Using-CT-Scanned-Image/tree/master (accessed on 1 November 2024)) (3956 images, four classes), Breast Tumor (https://www.kaggle.com/datasets/sabahesaraki/breast-ultrasound-images-dataset (accessed on 1 November 2024)) (780 images, three classes), Cataract (https://www.kaggle.com/datasets/jr2ngb/cataractdataset (accessed on 1 November 2024)) (601 images, four classes), and PAPILA (https://figshare.com/articles/dataset/PAPILA/14798004?file=35013982 (accessed on 1 November 2024)) (488 images, three classes). The Multiple Myeloma dataset was retrospectively collected at the University of Michigan, whereas the remaining datasets were publicly available benchmarks. The auxiliary relation used by SRA was constructed according to the availability of medical prior information. When suitable medical prior information was available, the corresponding prior representation was used to compute the pairwise auxiliary relation. Otherwise, the auxiliary relation was derived from the normalized Euclidean distance between the corresponding preprocessed input images. The same construction rule was applied consistently to all samples within each dataset. Table 1 summarizes the information source and construction strategy used for all nine datasets. Notably, all these public datasets were accessed and downloaded in November 2024.

For each dataset, 40% of the data were used exclusively for teacher pre-training. The remaining 60% were reserved for downstream model development and evaluation and were further divided into training, validation, and test subsets at a ratio of 70:10:20, corresponding to 42%, 6%, and 12% of the full dataset, respectively. Notably, the partitioning strategy accounted for related images within individual datasets to avoid cross-partition overlap. HAM10000 was split at the lesion level, whereas PAPILA and Breast Tumor were split at the patient level, ensuring that images from the same lesion or patient remained within the same partition. For the institutionally collected Multiple Myeloma dataset, all images from the same patient were assigned to the same partition. For LC25000, each original source image and its augmentation-derived images were grouped and assigned to the same partition. For the remaining public datasets, partitioning followed the independent sampling units supported by the available dataset structure and metadata. We additionally evaluated a conventional KD protocol in which the original teacher-pretraining subset and downstream training subset were combined to form a shared training partition comprising 82% of the full dataset. Both the teacher and student were trained using this shared partition, while the original validation and test subsets, comprising 6% and 12% of the full dataset, respectively, were retained without modification. Results from the two protocols are reported separately. The same partitioning strategy was applied to all comparison methods to ensure a consistent experimental protocol. In the principal non-overlapping protocol, SCOPE and all competing KD methods used only the training subset derived from the downstream 60% partition. The non-KD classification baselines adopted the same downstream training, validation, and test subsets. All principal experiments were repeated using five random seeds. The same data partitions and random seeds were used across the compared methods to enable matched comparisons. For each method, model selection was performed independently in each run using the highest validation ACC. The results are reported as the mean ± standard deviation across the five runs.

#### 4.1.2. Comparison Methods

SCOPE was compared with two standard classification baselines, representative knowledge distillation methods, and additional learning-based comparison approaches. Baseline-1 and Baseline-2 used ResNet18 and ResNet101 [34], respectively, and were trained from scratch using identical data partitions and optimization settings. The knowledge distillation methods included USKD [35], LSKD [13], OFAKD [36], UniDistill [37], SDKD [8], MCAD-KD [18], LDRLD [12], FoPro-KD [38], and KD-FMV [9]. Additional comparison approaches included PETL [3], AFA [39], DEPICT [40], ProtoPNets [41], and Diff-Mix [42]. All methods were evaluated using identical data partitions whenever applicable. The default teacher and student architectures were ResNet101 and ResNet18, respectively. Additional experiments using ViT-B and WideResNet101 as teachers and ShuffleNetV2 as an alternative student were conducted to assess generalization across heterogeneous teacher–student architectures.

#### 4.1.3. Implementation Details

The final hyperparameter configuration and the corresponding selection strategies are summarized in Table 2. All input images were resized to 512×512 pixels and normalized using the ImageNet mean and standard deviation. Training was performed for 100 epochs using the Adam optimizer with a batch size of 64 and a learning rate of $1\times 10^{-4}$. For each run, the model checkpoint achieving the highest validation ACC was selected for test-set evaluation. The test set was not used for hyperparameter tuning, checkpoint selection, or any other model-development decision.

#### 4.1.4. Evaluation Metrics

Classification performance was evaluated using accuracy (ACC), macro-averaged F1 score (Macro-F1), one-vs-one area under the receiver operating characteristic curve (AUC-OVO), and one-vs-rest AUC (AUC-OVR). Computational efficiency was evaluated using the number of trainable parameters, floating-point operations (FLOPs), and inference latency on CPU and GPU platforms. Higher values indicate better performance for ACC, Macro-F1, AUC-OVO, and AUC-OVR, whereas lower values indicate greater efficiency for model size, FLOPs, and inference latency. Statistical comparisons between SCOPE and each competing method were performed using two-sided paired *t*-tests based on the five seed-matched runs. Statistical significance was defined as p<0.05. To account for multiple comparisons, Holm correction was applied separately within each dataset–metric combination across all pairwise comparisons between SCOPE and the competing methods, and the resulting adjusted *p*-values are reported in Table 3. To empirically examine whether teacher entropy reflects prediction reliability, test samples were grouped into three intervals according to their normalized teacher entropy, $H(p_{i}^{t})/\log C$: [0,0.33), [0.33,0.66), and [0.66,1.0]. Model calibration was evaluated using the expected calibration error: $\mathrm{ECE}=\sum_{b=1}^{M}\frac{|B_{b}|}{n}\left|\mathrm{acc}\left(B_{b}\right)-\mathrm{conf}\left(B_{b}\right)\right|.$ Here, $B_{b}$ denotes the set of test predictions assigned to confidence bin *b*, and lower ECE indicates better calibration. Uncertainty reliability was assessed by treating incorrect predictions as positive cases and computing the area under the ROC curve for error detection (error-detection AUC), using predictive entropy as the detection score. Robustness was evaluated under a controlled simulated perturbation by adding Gaussian noise with standard deviation σ=0.05 to the test images and measuring the resulting change in classification performance. This experiment evaluates sensitivity to the specified synthetic perturbation and is not interpreted as validation under real scanner-, protocol-, or institution-related acquisition shifts.

### 4.2. Overall Classification Performance

Table 4 summarizes the classification results across the nine datasets. SCOPE achieved the highest ACC among the two standard classification baselines on all nine datasets and maintained competitive or superior performance across most metrics. Relative to Baseline-2, SCOPE improved mean ACC by 3.8 percentage points on LC25000 (98.3% vs. 94.5%), 5.2 points on HAM10000 (84.0% vs. 78.8%), 17.0 points on Cataract (73.0% vs. 56.0%), and 19.0 points on PAPILA (82.9% vs. 63.9%). Compared with MCAD-KD on LC25000, SCOPE improved mean ACC and Macro-F1 by 5.7 and 6.5 percentage points, respectively. On Brain Tumor, SCOPE exceeded OFAKD by 9.0 points in ACC and 8.2 points in Macro-F1. On Multiple Myeloma, SCOPE achieved 64.1% ACC and 65.0% Macro-F1, exceeding KD-FMV by 5.0 and 5.6 points, respectively, while its AUC-OVO and AUC-OVR remained competitive. Although SCOPE did not obtain the highest value for every individual metric, it showed strong and comparatively stable performance across datasets of different scales. Table 3 further reports the paired statistical comparisons for each dataset–metric combination without collapsing the results into a single aggregate claim. The results show that the performance gains of SCOPE are statistically supported for a substantial number of comparisons, particularly for ACC and Macro-F1, although the significance varies across datasets, metrics, and competing methods. This pattern is consistent with the heterogeneous performance differences observed in Table 4: large improvements generally lead to stronger statistical evidence, whereas smaller differences or competitive AUC results are less consistently significant after Holm correction. The ablation and optimization-strategy comparisons are further summarized in Table 5. Table 6 evaluates calibration, error-detection ability, and robustness on three representative datasets. SCOPE consistently achieves lower ECE than KD-FMV on Chaoyang, Brain Tumor, and Breast Tumor, indicating better calibrated predictions, while its higher ED-AUC suggests that predictive entropy more effectively distinguishes erroneous predictions. SCOPE also maintains higher accuracy under the specified Gaussian-noise perturbation on all three datasets; for example, on Breast Tumor, the noisy accuracy increases from 72.8% for KD-FMV to 82.1% for SCOPE. Together, these results suggest that the benefits of SCOPE extend beyond clean classification accuracy to prediction calibration, error awareness, and robustness under the evaluated synthetic perturbation. These analyses are restricted to the three evaluated datasets and to the KD-FMV comparison and therefore should not be generalized to all competing methods or to real-world acquisition shifts.

### 4.3. Ablation and Protocol Analysis

Table 5 summarizes the ablation and optimization-strategy comparisons. For the SRA-related ablations, No SRA & No GCR removes both proposed components while retaining the standard classification and KD objectives; Teacher-Only uses only the teacher-derived relation; Label-Only retains only ground-truth classification supervision; No-Prior removes the auxiliary relation; Fixed-Alpha (0.50) replaces the confidence-derived pairwise weight with a constant $\alpha_{ij}^{t}=0.5$; and MaxProb-Alpha constructs the pairwise weight from the product of the maximum teacher class probabilities of the two samples. For the GCR-related ablations, No GCR removes gradient correction, while PCGrad and CAGrad replace GCR with the corresponding gradient-conflict optimization strategies. Alternative correction thresholds were also evaluated. To examine the effect of replacing the hard correction with continuous weighting, we additionally implemented a Soft GCR variant. Soft GCR continuously interpolates between the original distillation gradient and its fully projected counterpart according to the degree of gradient conflict. Specifically, the projection weight is defined as: w(ρ)=max(0,−ρ), and the corrected distillation gradient is given by: ${\tilde{g}}_{\mathrm{KD}}^{\mathrm{soft}}=g_{\mathrm{KD}}-w\left(\rho \right)\frac{\langle g_{\mathrm{KD}},g_{\mathrm{CE}}\rangle }{{∥g_{\mathrm{CE}}∥}_{2}^{2}+\epsilon }g_{\mathrm{CE}}$. No correction is applied when ρ≥0, whereas the correction strength increases continuously as the cosine similarity becomes more negative. Soft GCR was evaluated using the same training settings and computational cost as hard GCR.

Removing both SRA and GCR reduced mean Chaoyang ACC and Macro-F1 from 82.8% and 78.9% to 77.5% and 73.2%, respectively. The Teacher-Only, No-Prior, Fixed-Alpha, and MaxProb-Alpha variants also showed inferior overall performance, supporting the use of confidence-aware integration of teacher relations and auxiliary priors. The No-Prior variant generally showed lower overall performance than the complete SCOPE framework. In this setting, the auxiliary prior branch is removed, so that $R_{\mathrm{ref}}^{ij}=R_{t}^{ij}$ and SRA reduces to teacher-based relational distillation. These results show that SCOPE remains applicable even when neither metadata-based nor image-derived prior information is used.

**Figure 2:**
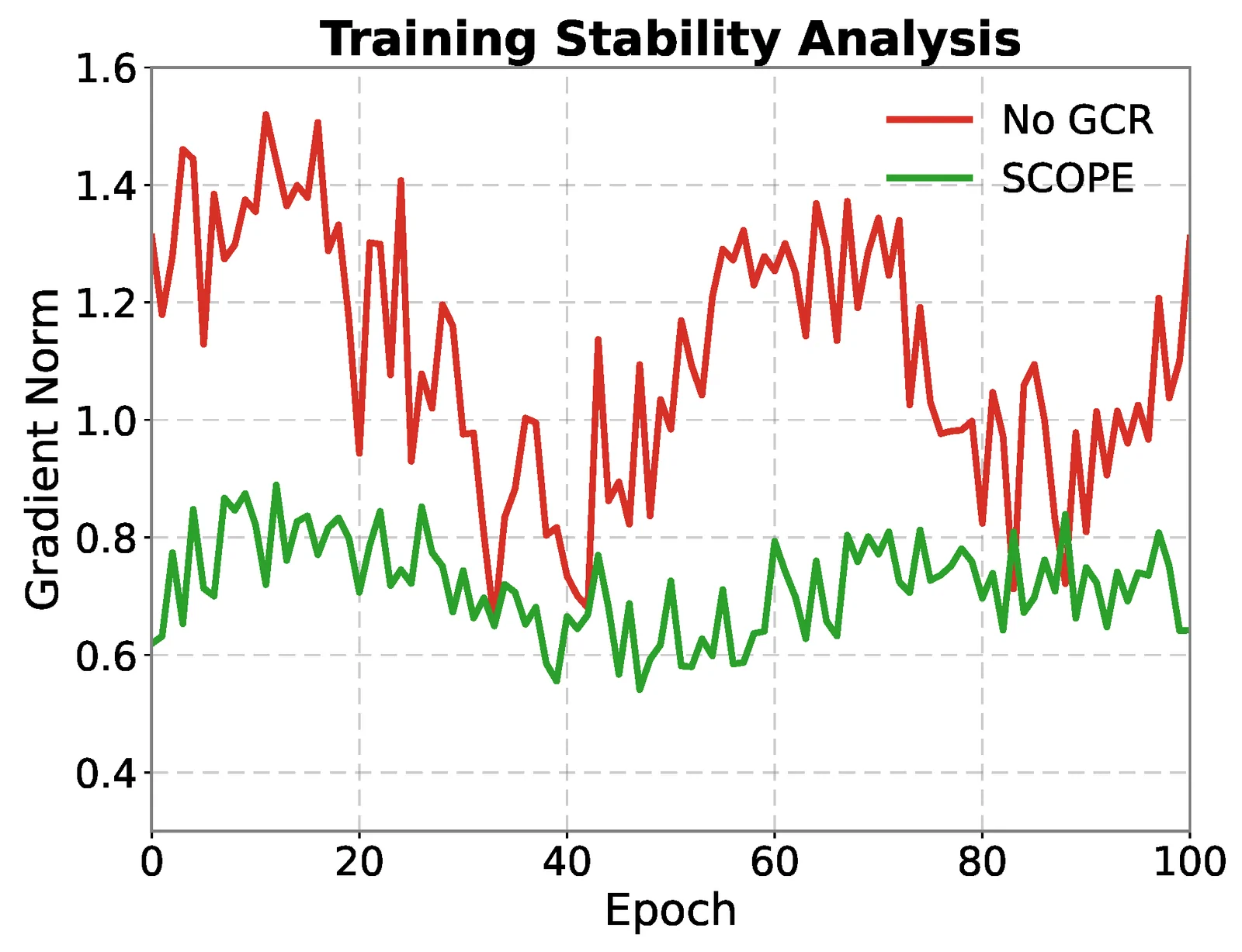
Comparison of total gradient norm during training on the LC25000 dataset.

**Figure 3:**
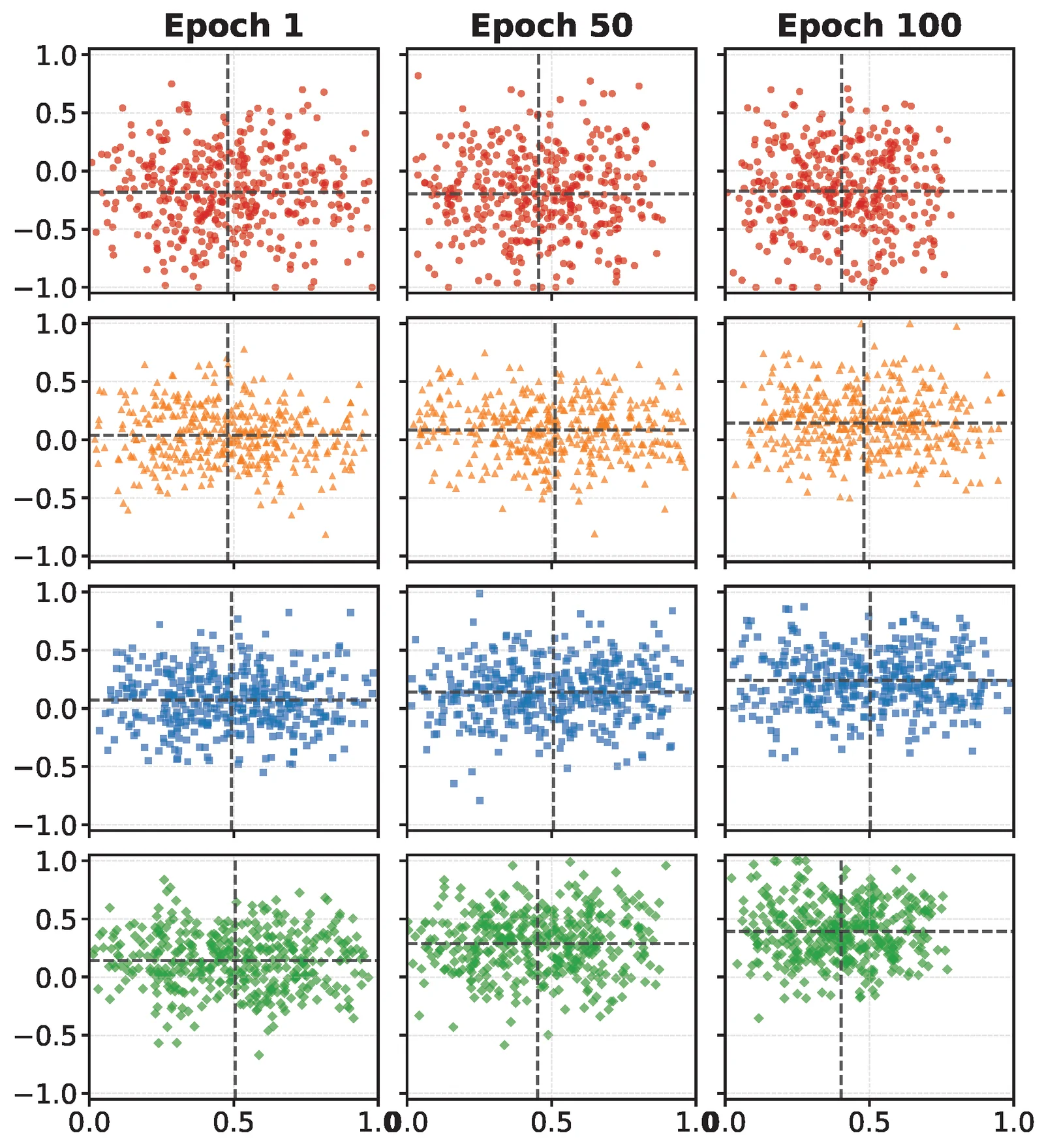
Visualization of the entropy–gradient similarity distributions on the Multiple Myeloma dataset. Each row corresponds to a different optimization strategy (No GCR, PCGrad, CAGrad, and GCR, from top to bottom), and each column corresponds to a training epoch (1, 50, and 100, from left to right). The horizontal axis denotes the entropy of the teacher predictions, with lower values corresponding to higher confidence, while the vertical axis indicates the cosine similarity between the classification and distillation gradients.

The hard-GCR, Soft-GCR, No-GCR, PCGrad, CAGrad, and alternative-threshold controls are evaluated under the same architecture and data protocol. The continuous control uses the weighting rule defined above, whereas hard GCR applies the full projection whenever ρ<0. The threshold analysis further shows that the relative performance varies across datasets and evaluation metrics. Although some alternative thresholds perform favorably for individual dataset–metric combinations, none provides a consistent advantage across the evaluated settings, while δ=0 maintains competitive overall performance. Additionally, this fixed threshold also avoids additional dataset-specific threshold tuning, thereby reducing the tuning cost. This observation is consistent with our use of δ=0 as a geometrically defined default rather than a threshold selected through dataset-specific empirical tuning. Figure 2 illustrates the gradient-norm trajectories, and Figure 3 visualizes the entropy–gradient-similarity distributions.

To examine whether the observed performance gains were primarily attributable to the non-overlapping teacher-pretraining and student-training partitions, we additionally evaluated vanilla KD and SCOPE under a conventional KD protocol in which the teacher and student shared the same training partition. As shown in Table 5, SCOPE maintained a numerical advantage over vanilla KD across the three evaluated datasets under this standard protocol. Specifically, SCOPE achieved higher ACC and Macro-F1 on Chaoyang, Brain Tumor, and Breast Tumor, with corresponding improvements also observed for most AUC metrics. These results suggest that the performance gains of SCOPE are not solely attributable to the original non-overlapping data partition. However, because this control experiment was conducted on only three datasets, broader conclusions regarding protocol independence require further evaluation on additional datasets.

### 4.4. Relationship Between Teacher Entropy and Classification Accuracy

The teacher classification accuracy was then calculated within each entropy interval, separately for each dataset. Table 7 reports the resulting teacher accuracy across the three normalized entropy intervals for all nine datasets. A consistent monotonic pattern was observed: lower-entropy samples exhibited higher teacher accuracy, whereas accuracy decreased as entropy increased. For example, teacher accuracy on Chaoyang decreased from 91.43% in [0,0.33) to 78.85% in [0.33,0.66) and 58.64% in [0.66,1.0]. A similar trend was observed on LC25000 (98.48%, 92.37%, and 77.15%, respectively). These results provide empirical support for using normalized teacher entropy as a relative reliability indicator in SRA.

### 4.5. Effect of Additional Training Data

Baseline-2 was trained using only the downstream training subset, whereas Baseline-2 (Expanded Training Set) additionally incorporated the subset used for teacher pre-training. Relative to the original Baseline-2, expanding the training set increased ACC from 76.1% to 80.9% on Chaoyang, from 78.9% to 80.8% on Brain Tumor, and from 56.0% to 71.6% on Breast Tumor. These results confirm that the additional training data improved the high-capacity baseline across the three evaluated datasets. Despite this expanded training set, SCOPE remained competitive with or outperformed Baseline-2 (Expanded Training Set) in ACC. SCOPE achieved 82.8 ± 1.8% ACC on Chaoyang, compared with 80.9 ± 2.8% for the expanded baseline, and 82.8 ± 2.0% on Brain Tumor, compared with 80.8 ± 2.5%. On Breast Tumor, the difference was larger, with SCOPE achieving 85.3 ± 1.1% ACC compared with 71.6 ± 2.1%. These results indicate that the performance advantage of SCOPE cannot be explained solely by differences in the amount of training data available to the baseline.

### 4.6. Computational Efficiency

Table 8 summarizes the computational efficiency results. Using the five-run results from the principal evaluation, SCOPE achieved an ACC of 98.3±1.5% on LC25000 with 11.19 million parameters and 9.69 GFLOPs. Its GPU and CPU inference latencies were 0.034 s and 0.081 s per sample, respectively. Baseline-2 required 44.50 million parameters and 40.12 GFLOPs and achieved an ACC of 94.5±2.9%. Several higher-complexity methods could not be executed successfully in the CPU-only environment. Under the controlled evaluation protocol, SCOPE provided a favorable balance between classification performance and computational cost.

### 4.7. Generalization Across Teacher–Student Architectures

SCOPE maintained strong performance across ResNet101, WideResNet101, and ViT-B teachers and ResNet18 and ShuffleNetV2 students. The results in Table 9 show that the ViT-B→ResNet18 and WideResNet101→ResNet18 configurations achieved LC25000 ACC values of 99.8% and 99.7%, respectively, while the default ResNet101→ResNet18 configuration achieved 98.3%. The ShuffleNetV2 student also retained competitive performance across various medical imaging datasets. These complementary architecture-generalization experiments assess the portability of SCOPE when either the teacher or student backbone is changed. Although the optimal configuration varied by dataset and metric, the results indicate that SCOPE is not restricted to a single teacher–student architecture.

### 4.8. Hyperparameter Configuration and Sensitivity

The sensitivity results are summarized in Table 10. SCOPE maintained relatively stable performance over moderate ranges of the evaluated hyperparameters. For β, the strongest overall performance was observed within approximately 0.5–1.5, with β=1.0 achieving 82.8% ACC and 80.0% Macro-F1. For γ, performance remained competitive within 0.8–1.5, while γ=0.8 yielded the highest ACC of 83.2%. For λ, the results were generally stable between 0.5 and 1.0, with λ=0.8 providing a favorable balance across the four evaluation metrics. Similarly, the temperature showed its strongest performance in the range T=1.5–2.5, with T=2.0 achieving 82.8% ACC and 80.0% Macro-F1. These results indicate that the selected configuration lies within a relatively stable performance region rather than at an isolated metric-specific optimum.

## 5. Discussion

The results demonstrate that SCOPE can improve the performance of lightweight student models across medical imaging datasets of different sizes while maintaining relatively low computational complexity. These findings suggest that selective knowledge transfer may be especially useful when the amount of student training data is limited. The ablation results further clarify how SCOPE controls the influence of teacher knowledge during student learning. SRA adjusts the contribution of teacher-derived relations according to pairwise confidence, whereas GCR intervenes only when the distillation gradient conflicts with the ground-truth classification gradient. This allows teacher information to be retained when it is compatible with the supervised objective, while limiting its influence when local conflicts arise during optimization. Although some variants achieved strong results on individual metrics, the complete SCOPE framework showed more consistent overall performance.

SCOPE also remained effective across different teacher–student architectures and retained a computational footprint comparable to that of the lightweight student model. These findings indicate potential utility in medical imaging applications with limited computational resources. However, the reported latency values were obtained under a controlled offline setting on a single hardware configuration and should not be interpreted as evidence of clinical deployment readiness. This study has several limitations. The non-overlapping teacher and student data partitions differ from the conventional KD setting, and any conclusion from the standard-protocol control must remain restricted to the datasets actually evaluated. The auxiliary relation is dataset-dependent because its construction relies on the type of medical prior information available for each dataset. When suitable medical prior information is available, the relation is derived from the corresponding prior representation; otherwise, a normalized Euclidean image-based distance is used. Although this strategy allows SCOPE to operate across datasets with heterogeneous prior availability, the informativeness of the auxiliary relation may vary across datasets and imaging modalities. The auxiliary relation is used only during training, and the No-Prior control removes this component while retaining teacher-based relational distillation.

## 6. Conclusions

We formulated SCOPE as a confidence- and conflict-aware knowledge distillation framework that integrates confidence-aware relational distillation with classification-priority gradient correction. SCOPE regulates unreliable teacher supervision at the relational level while mitigating conflicting KD gradients at the optimization level. Experiments across nine medical image classification datasets and multiple teacher–student architectures demonstrated competitive predictive performance with relatively low computational overhead. These findings support the potential of SCOPE for resource-constrained medical image analysis, although further validation using independent clinical cohorts and additional teacher–student architectures is warranted.

## Figures and Tables

**Table 1:** Dataset-specific construction of the auxiliary relation used in SRA. When suitable medical prior information is available, the auxiliary relation is constructed from the normalized Euclidean distance between prior representations; otherwise, it is constructed from the normalized Euclidean distance between preprocessed input images.

Dataset	Auxiliary Relation	Information Used	Prior Dimension	Construction
LC25000	Image-based	Input images	–	Normalized Euclidean image distance
HAM10000	Prior-based	Age; gender	3	Standardization/one-hot encoding
Chaoyang	Image-based	Input images	–	Normalized Euclidean image distance
Multiple Myeloma	Image-based	Input images	–	Normalized Euclidean image distance
Brain Tumor	Image-based	Input images	–	Normalized Euclidean image distance
Kidney	Image-based	Input images	–	Normalized Euclidean image distance
Breast Tumor	Image-based	Input images	–	Normalized Euclidean image distance
Cataract	Image-based	Input images	–	Normalized Euclidean image distance
PAPILA	Prior-based	Age; gender	3	Standardization/one-hot encoding

Note: For datasets with suitable medical prior information, each sample is represented by a prior vector $m_{i}\in R^{d_{r}}$. Continuous variables are standardized using statistics estimated exclusively from the corresponding training partition, whereas categorical variables are encoded using one-hot representations. The pairwise auxiliary relation is then constructed from the normalized Euclidean distance $d_{m}\left(m_{i},m_{j}\right)$. For datasets without suitable medical prior information, the auxiliary relation is instead derived directly from the normalized Euclidean image distance $d_{x}\left(x_{i},x_{j}\right)$. The same construction rule is applied consistently to all samples within each dataset, and no additional trainable network is introduced for constructing the auxiliary relation. For image-based auxiliary relations, no separate prior representation is constructed; the normalized Euclidean image distance is computed directly from the vectorized preprocessed images. Regardless of the information source, the resulting pairwise auxiliary relation $R_{ij}^{o}$ is scalar-valued. For HAM10000 and PAPILA, age and gender were obtained from the metadata provided with the corresponding datasets. No missing values were present in these attributes; therefore, no attribute imputation was required.

**Table 2:** Summary of the hyperparameters, training settings, and selection strategies used in the main experiments.

Parameter or Setting	Description	Value
β	Weight of the classification loss	1.0
γ	Scaling coefficient for the KD loss	1.0
λ	Weight of the corrected KD gradient	0.8
*T*	Distillation temperature	2.0
τ	Decay parameter for the auxiliary relation	1.0
δ	Gradient-conflict threshold	0
Optimizer	Optimization algorithm	Adam
Learning rate	Initial learning rate	$1\times 10^{-4}$
Learning-rate schedule	Learning-rate adjustment strategy	Fixed learning rate
Batch size	Number of samples per mini-batch	64
Number of epochs	Maximum training duration	100
Checkpoint selection	Criterion for selecting the final model	Highest validation ACC

**Table 3:** Paired statistical comparisons between SCOPE and all competing methods across the nine datasets. Each entry reports the Holm-adjusted *p*-value from a two-sided paired *t*-test based on five seed-matched runs. Holm correction was applied separately within each dataset–metric combination across all comparisons with SCOPE. Values below 0.001 are reported as <0.001.

**Method**	**LC25000**				**HAM10000**				**Chaoyang**			
	**ACC**	**Macro-F1**	**AUC-OVO**	**AUC-OVR**	**ACC**	**Macro-F1**	**AUC-OVO**	**AUC-OVR**	**ACC**	**Macro-F1**	**AUC-OVO**	**AUC-OVR**
Baseline-1	<0.001	<0.001	0.003	0.026	0.002	<0.001	0.002	0.004	<0.001	<0.001	<0.001	<0.001
Baseline-2	0.185	0.129	1.000	1.000	0.118	0.003	0.017	0.069	0.022	0.013	0.021	0.088
USKD	0.001	<0.001	0.016	0.151	0.002	<0.001	<0.001	<0.001	0.003	<0.001	0.091	0.165
LSKD	0.273	0.129	0.690	1.000	0.061	<0.001	0.009	0.026	0.011	<0.001	0.014	0.069
OFAKD	<0.001	<0.001	0.073	0.117	0.033	0.002	0.004	0.026	0.001	<0.001	0.012	<0.001
UniDistill	0.018	0.003	1.000	1.000	0.029	0.001	0.005	0.018	0.021	0.014	0.091	0.444
SDKD	0.185	0.129	1.000	1.000	0.161	0.076	0.241	1.000	0.027	0.019	0.275	0.840
PETL	0.017	0.010	1.000	1.000	0.069	<0.001	0.009	0.110	0.023	0.019	0.275	0.444
MCAD-KD	0.101	0.048	1.000	1.000	0.006	0.003	0.005	0.012	0.023	0.013	0.075	0.287
LDRLD	0.001	<0.001	0.649	1.000	0.065	0.003	0.275	0.242	0.007	0.003	0.021	0.137
FoPro-KD	0.005	0.002	0.065	0.086	0.069	0.001	0.007	0.069	<0.001	<0.001	0.017	0.002
KD-FMV	0.013	0.003	0.061	0.151	0.161	0.014	0.885	1.000	0.009	0.013	0.275	0.840
AFA	0.001	0.002	0.855	1.000	0.118	0.724	0.007	0.069	0.005	<0.001	0.002	0.492
DEPICT	<0.001	0.002	0.181	0.170	0.033	0.364	<0.001	<0.001	<0.001	<0.001	0.005	0.037
ProtoPNets	0.017	0.009	0.862	1.000	0.161	0.724	0.007	0.027	0.009	<0.001	0.021	0.214
Diff-Mix	<0.001	<0.001	0.016	0.006	0.015	0.006	0.005	0.027	0.027	0.019	0.239	0.492
**Method**	**Multiple Myeloma**				**Brain Tumor**				**Kidney**			
	**ACC**	**Macro-F1**	**AUC-OVO**	**AUC-OVR**	**ACC**	**Macro-F1**	**AUC-OVO**	**AUC-OVR**	**ACC**	**Macro-F1**	**AUC-OVO**	**AUC-OVR**
Baseline-1	0.012	0.014	0.029	0.006	0.211	0.340	0.536	0.672	0.023	0.001	0.529	0.256
Baseline-2	0.012	0.014	0.256	0.163	0.211	0.269	0.509	0.672	0.216	0.119	1.000	1.000
USKD	0.012	0.007	0.029	0.163	0.061	0.117	1.000	0.898	0.012	<0.001	0.354	0.491
LSKD	0.012	<0.001	0.020	0.062	0.211	0.340	1.000	1.000	0.010	0.012	1.000	1.000
OFAKD	0.007	0.017	1.000	1.000	0.024	0.023	1.000	0.964	0.010	<0.001	0.893	0.621
UniDistill	<0.001	<0.001	0.001	<0.001	0.069	0.198	1.000	0.112	0.290	0.119	1.000	1.000
SDKD	0.029	0.022	0.374	0.175	0.211	0.198	1.000	1.000	0.140	0.008	0.427	0.946
PETL	0.029	0.022	1.000	1.000	<0.001	<0.001	1.000	0.223	0.023	0.004	1.000	1.000
MCAD-KD	0.012	0.003	0.374	1.000	0.002	0.002	1.000	0.493	0.071	0.035	1.000	0.843
LDRLD	0.012	0.014	0.256	0.137	0.039	0.069	1.000	0.603	0.290	0.158	1.000	1.000
FoPro-KD	0.005	0.009	0.256	0.005	0.040	0.076	1.000	1.000	0.140	0.025	1.000	1.000
KD-FMV	0.012	0.014	1.000	1.000	0.211	0.198	1.000	0.769	0.071	0.008	1.000	1.000
AFA	0.034	0.040	0.025	0.096	0.040	0.095	1.000	1.000	0.035	0.006	0.893	0.867
DEPICT	0.012	0.008	0.134	0.175	0.057	0.141	1.000	1.000	0.007	0.001	1.000	1.000
ProtoPNets	0.029	0.008	0.034	0.383	0.202	0.340	0.058	0.815	0.014	0.003	1.000	1.000
Diff-Mix	0.029	0.040	0.374	0.175	0.119	0.198	0.483	0.451	<0.001	0.002	1.000	1.000
**Method**	**Breast Tumor**				**Cataract**				**PAPILA**			
	**ACC**	**Macro-F1**	**AUC-OVO**	**AUC-OVR**	**ACC**	**Macro-F1**	**AUC-OVO**	**AUC-OVR**	**ACC**	**Macro-F1**	**AUC-OVO**	**AUC-OVR**
Baseline-1	0.003	0.001	0.009	0.026	0.002	0.008	0.004	0.031	0.002	0.005	0.028	0.032
Baseline-2	<0.001	<0.001	<0.001	<0.001	<0.001	<0.001	<0.001	<0.001	<0.001	<0.001	0.062	0.067
USKD	0.005	0.012	0.109	0.093	0.002	<0.001	0.002	0.001	0.002	0.006	0.062	0.462
LSKD	<0.001	<0.001	0.109	0.083	0.011	0.001	0.013	0.030	<0.001	<0.001	0.012	1.000
OFAKD	<0.001	<0.001	0.002	0.010	0.086	0.894	0.233	0.106	0.002	<0.001	0.002	0.047
UniDistill	0.002	0.002	0.109	0.083	0.002	<0.001	<0.001	<0.001	<0.001	<0.001	<0.001	<0.001
SDKD	0.037	0.287	0.120	0.303	0.039	0.049	0.408	0.106	0.016	0.030	0.012	0.045
PETL	0.002	0.012	0.019	0.003	<0.001	<0.001	<0.001	<0.001	0.009	0.003	0.054	0.224
MCAD-KD	<0.001	<0.001	0.015	0.006	0.011	0.011	0.205	0.100	<0.001	<0.001	<0.001	0.013
LDRLD	0.004	0.012	0.120	0.303	0.002	<0.001	0.002	0.002	0.001	<0.001	<0.001	<0.001
FoPro-KD	<0.001	<0.001	0.037	0.033	0.002	0.002	0.047	0.031	0.002	<0.001	<0.001	0.001
KD-FMV	0.001	<0.001	0.120	0.416	0.003	0.011	0.176	0.031	0.002	<0.001	0.003	0.013
AFA	0.019	0.041	0.120	0.083	0.004	0.001	0.028	0.004	0.016	0.016	0.089	0.462
DEPICT	0.003	0.012	0.003	0.076	0.002	0.010	0.086	0.060	0.022	0.016	0.054	1.000
ProtoPNets	0.001	0.003	0.010	0.006	0.011	0.002	0.057	0.038	0.016	0.007	<0.001	0.002
Diff-Mix	0.019	0.055	0.120	0.082	0.003	0.010	0.205	0.100	0.016	0.016	0.004	0.020

Note: Two-sided paired *t*-tests were performed between each competing method and SCOPE using five seed-matched runs. Holm correction was applied separately within each dataset–metric combination across the 16 comparisons with SCOPE to control the family-wise error rate. Each entry reports the Holm-adjusted *p*-value. Values below 0.001 are reported as <0.001.

**Table 4:** Comparison with various methods across different classification tasks (%). Results are reported as the mean ± standard deviation over 5 matched runs. The best results are underlined.

**Method**	**LC25000**				**HAM10000**				**Chaoyang**			
	**ACC**	**Macro-F1**	**AUC-OVO**	**AUC-OVR**	**ACC**	**Macro-F1**	**AUC-OVO**	**AUC-OVR**	**ACC**	**Macro-F1**	**AUC-OVO**	**AUC-OVR**
Baseline-1	78.9±2.3	76.0±2.4	90.0±2.1	90.1±2.5	73.3±2.3	51.3±2.1	84.1±2.4	86.7±2.6	61.2±1.3	36.5±1.5	78.1±1.3	81.6±1.1
Baseline-2	94.5±2.9	94.6±2.9	97.7±2.7	97.7±2.7	78.8±2.4	54.6±2.3	85.2±2.3	89.2±1.8	76.1±1.1	69.4±1.3	86.5±0.7	88.4±1.6
USKD	82.5±2.0	82.9±2.1	94.7±1.7	94.6±2.1	72.3±2.5	40.2±2.3	70.7±2.5	82.2±2.3	72.5±2.7	64.8±2.3	87.4±2.7	88.3±2.6
LSKD	97.1±1.3	96.9±1.4	96.8±1.2	97.5±1.4	77.7±2.5	46.4±2.7	81.3±2.6	86.2±2.5	74.3±2.4	57.7±2.5	82.1±2.4	86.1±2.7
OFAKD	83.2±1.8	82.8±1.7	94.8±1.9	95.0±1.8	76.2±2.3	54.1±2.0	81.1±2.3	86.7±2.5	69.8±1.6	65.0±1.6	83.4±1.6	84.0±1.6
UniDistill	93.2±1.0	93.1±0.8	98.5±0.9	98.6±1.0	76.2±1.4	51.6±1.3	80.6±1.5	86.7±1.4	77.4±2.3	72.1±2.5	88.0±2.5	89.9±2.6
SDKD	96.0±1.5	96.1±1.5	99.0±1.1	98.4±1.4	81.4±0.8	65.4±0.9	90.1±0.7	94.3±1.0	76.7±2.5	72.5±2.3	90.4±2.5	91.6±2.7
PETL	91.6±1.4	91.4±1.6	97.4±1.7	97.2±1.7	76.5±2.1	39.8±1.9	80.5±2.2	89.4±1.9	77.1±2.3	72.5±2.3	89.4±2.6	90.5±2.5
MCAD-KD	92.6±2.4	92.2±2.5	98.5±2.0	98.7±1.8	77.2±1.8	59.4±1.6	85.4±1.9	89.2±2.1	74.3±2.8	67.2±2.6	87.6±3.1	89.2±2.8
LDRLD	84.4±2.4	84.3±2.3	96.7±2.4	96.6±2.3	78.4±2.6	57.1±2.8	90.4±2.7	93.0±2.6	74.0±1.9	66.3±2.1	87.0±1.5	88.5±1.9
FoPro-KD	90.1±1.9	89.9±1.9	94.5±2.0	94.7±1.7	76.6±2.7	44.0±2.6	77.1±2.6	86.8±2.7	66.5±1.5	59.3±1.6	83.6±1.8	84.0±1.4
KD-FMV	91.0±2.1	90.8±2.0	95.2±2.2	95.5±2.1	79.0±2.6	56.7±2.9	92.4±2.4	94.3±2.4	77.7±1.2	73.3±1.3	91.5±1.6	92.3±1.2
AFA	84.2±1.3	83.5±1.7	97.3±1.3	97.7±1.4	80.0±0.6	69.8±0.4	82.4±1.1	89.9±0.6	74.2±2.0	65.7±1.9	88.0±1.8	90.6±2.3
DEPICT	92.2±1.2	92.1±1.2	97.0±1.1	96.7±1.7	75.9±2.5	68.2±2.3	63.9±2.6	61.3±2.6	73.6±1.2	64.5±1.4	85.8±1.2	87.9±1.4
ProtoPNets	91.1±1.7	91.1±1.4	96.7±1.9	96.7±1.5	79.7±2.3	72.9±2.2	78.0±2.2	85.0±2.0	73.4±2.3	59.1±2.7	86.0±2.5	88.5±2.4
Diff-Mix	84.7±1.3	83.2±1.9	96.0±1.5	95.9±1.0	76.7±1.1	63.2±1.4	84.3±1.2	89.1±1.0	77.7±1.8	72.5±1.5	89.8±1.7	91.3±1.6
SCOPE	98.3±1.5	98.7±1.3	98.9±1.3	98.5±1.4	84.0±2.3	71.0±2.6	92.7±2.1	95.1±2.5	82.8±1.8	78.9±2.0	92.9±1.8	92.7±1.3
**Method**	**Multiple Myeloma**				**Brain Tumor**				**Kidney**			
	**ACC**	**Macro-F1**	**AUC-OVO**	**AUC-OVR**	**ACC**	**Macro-F1**	**AUC-OVO**	**AUC-OVR**	**ACC**	**Macro-F1**	**AUC-OVO**	**AUC-OVR**
Baseline-1	51.3±3.0	49.5±2.9	71.0±3.0	70.2±2.6	78.9±2.3	78.2±2.7	88.4±2.2	88.6±2.2	93.0±1.7	89.1±1.8	96.7±1.8	96.6±1.7
Baseline-2	56.5±2.4	54.9±2.0	77.2±2.4	76.9±2.5	78.9±1.6	77.9±1.8	88.8±1.6	89.0±1.7	95.7±2.1	95.1±2.0	97.7±2.0	98.2±1.7
USKD	53.1±2.1	53.3±2.0	77.9±2.0	77.3±2.1	77.2±1.1	76.0±1.4	90.5±1.0	90.7±0.8	87.3±2.0	67.4±2.5	95.6±2.0	96.8±1.7
LSKD	54.6±0.8	44.9±1.0	74.7±0.8	75.1±1.1	79.3±2.4	78.8±2.4	91.6±2.5	91.4±2.4	91.5±1.8	89.1±2.1	97.4±1.7	97.4±1.9
OFAKD	57.1±1.8	56.4±2.4	80.9±2.0	80.4±1.8	73.8±2.6	73.3±2.7	90.2±2.5	90.5±2.2	89.1±1.6	86.3±1.5	96.8±2.1	97.3±1.6
UniDistill	51.1±0.9	37.0±0.5	60.2±1.3	60.3±1.3	80.0±2.4	79.2±2.3	89.0±2.4	89.4±2.3	97.2±0.8	96.2±0.9	98.5±1.1	98.2±0.9
SDKD	56.3±1.9	56.0±1.9	79.3±2.0	78.1±1.7	79.7±1.9	78.2±1.9	92.0±2.3	91.3±1.7	94.6±2.2	93.3±2.1	97.6±1.8	97.3±2.4
PETL	56.9±1.5	56.6±1.6	81.8±1.3	81.3±1.7	71.9±1.5	70.3±1.5	90.0±1.4	89.9±1.3	93.2±2.3	91.8±2.1	98.2±2.0	98.3±2.1
MCAD-KD	55.9±1.0	55.6±1.0	81.1±1.1	80.4±0.9	69.1±1.6	68.9±1.7	89.3±1.6	88.9±2.0	95.0±2.7	94.0±2.6	97.8±2.0	97.5±2.3
LDRLD	53.1±2.4	51.6±2.3	77.7±2.2	77.0±2.4	76.5±1.6	76.2±1.7	89.8±1.7	89.8±1.7	96.4±2.7	95.7±3.0	97.4±2.2	97.3±2.3
FoPro-KD	53.7±1.2	53.0±1.2	79.1±1.0	78.4±1.1	74.7±1.7	74.0±1.9	91.0±1.6	90.7±1.8	93.0±2.6	91.9±2.5	98.4±1.9	98.0±2.3
KD-FMV	59.1±1.2	59.4±1.1	81.9±1.4	81.2±1.2	80.0±2.0	78.3±2.3	90.1±2.1	90.7±1.9	95.1±1.9	94.1±1.7	97.7±1.7	98.1±1.8
AFA	58.8±2.0	59.1±1.9	77.5±2.0	76.8±2.0	76.5±1.8	76.4±1.9	91.1±1.8	91.2±2.0	90.8±2.2	88.3±2.4	96.7±2.0	96.7±2.2
DEPICT	59.2±1.9	59.5±1.7	77.6±1.9	76.7±2.3	77.3±1.8	77.6±1.8	92.7±1.9	92.3±1.7	83.7±2.5	79.6±2.5	97.3±2.3	97.7±2.1
ProtoPNets	58.2±1.7	58.2±1.9	79.0±1.9	78.2±1.7	80.2±0.9	79.8±0.8	94.5±0.8	94.5±0.9	87.7±2.0	84.8±2.2	97.3±2.0	97.6±2.1
Diff-Mix	59.1±2.0	59.3±1.8	78.7±1.9	77.7±2.1	79.8±2.3	79.1±2.3	94.1±2.2	94.4±2.1	81.9±1.6	83.0±1.8	96.7±1.7	97.0±1.5
SCOPE	64.1±2.0	65.0±2.4	82.4±1.9	80.7±1.2	82.8±2.0	81.5±2.0	90.9±1.4	92.5±2.0	98.5±1.4	98.9±1.3	98.8±1.4	99.2±1.3
**Method**	**Breast Tumor**				**Cataract**				**PAPILA**			
	**ACC**	**Macro-F1**	**AUC-OVO**	**AUC-OVR**	**ACC**	**Macro-F1**	**AUC-OVO**	**AUC-OVR**	**ACC**	**Macro-F1**	**AUC-OVO**	**AUC-OVR**
Baseline-1	74.3±2.2	68.9±2.2	84.2±2.1	84.9±2.4	65.7±1.5	59.0±1.7	78.7±1.5	79.4±1.5	78.4±0.9	66.1±0.6	80.7±0.7	80.0±0.6
Baseline-2	56.0±1.9	29.3±1.7	62.5±1.8	65.2±1.7	56.0±1.8	29.4±2.0	62.5±2.1	64.9±1.8	63.9±0.9	50.3±1.1	81.2±0.9	79.5±0.8
USKD	76.8±2.0	74.8±1.5	87.6±1.9	88.5±1.7	61.4±2.0	46.2±2.5	65.5±2.5	68.1±2.4	74.5±1.4	65.4±1.3	81.6±1.1	82.5±1.3
LSKD	64.8±1.8	46.4±2.0	87.7±1.8	88.5±1.4	61.6±2.5	42.0±1.9	72.4±2.2	74.0±2.3	71.3±1.8	44.7±2.0	79.6±2.2	83.5±2.1
OFAKD	73.9±0.9	68.3±1.2	83.2±0.9	85.2±1.1	70.0±2.4	66.0±2.4	82.0±2.5	80.8±2.7	70.3±2.2	42.6±1.9	73.9±2.1	79.2±1.9
UniDistill	72.4±2.3	66.1±2.3	87.5±2.4	87.6±2.1	63.0±0.7	39.4±0.8	57.2±1.1	60.0±0.8	67.7±1.6	26.8±1.1	49.7±1.3	50.8±1.5
SDKD	83.4±0.9	82.3±1.0	95.2±1.0	94.8±1.1	70.0±1.1	63.0±0.8	83.1±0.9	83.2±1.1	79.7±0.9	73.1±0.7	80.6±0.8	79.3±1.1
PETL	77.0±1.4	75.5±1.6	88.8±1.4	88.6±1.8	57.0±1.8	33.7±1.7	66.7±1.8	68.1±1.7	71.6±3.1	63.3±3.0	79.2±2.9	79.9±2.7
MCAD-KD	67.0±1.9	61.5±1.9	83.4±2.1	82.9±2.0	63.8±1.8	56.4±2.0	79.8±1.9	80.4±1.9	75.8±1.1	60.0±1.5	76.7±1.7	79.0±1.8
LDRLD	77.0±2.2	75.0±2.1	89.3±2.1	89.8±2.5	58.9±2.5	36.3±2.7	68.4±2.6	69.4±3.0	66.1±2.0	25.4±2.0	50.1±1.9	48.8±1.9
FoPro-KD	71.2±1.2	67.5±1.4	88.2±1.1	87.2±1.4	63.5±2.1	55.6±2.3	78.5±2.5	79.3±2.4	71.3±1.8	53.1±1.7	65.3±2.1	65.7±2.0
KD-FMV	79.2±1.9	77.3±1.9	92.3±2.1	92.2±1.8	66.8±1.5	60.7±1.9	80.9±2.0	80.2±1.9	72.3±2.1	61.2±1.9	76.0±1.9	77.5±1.8
AFA	79.0±1.8	75.5±2.5	88.5±2.2	89.0±1.9	66.5±1.1	56.1±1.0	78.3±1.1	77.9±0.5	76.1±2.6	66.0±2.6	81.1±2.8	81.1±2.4
DEPICT	79.6±1.6	78.0±2.0	89.0±1.6	89.1±2.2	59.7±1.8	57.2±2.1	80.2±2.0	80.1±1.9	79.7±1.4	70.8±1.0	82.1±0.9	83.7±1.3
ProtoPNets	76.8±1.7	73.4±1.4	86.7±1.9	87.5±1.6	63.2±1.8	48.8±1.6	78.5±1.5	78.5±1.7	79.7±1.8	70.7±1.5	77.5±1.6	78.0±1.9
Diff-Mix	80.4±1.6	78.8±2.2	89.5±1.6	88.8±1.7	62.2±2.5	57.2±2.2	79.9±2.8	80.8±2.9	78.1±2.2	69.2±2.0	74.9±2.3	77.4±2.3
**SCOPE**	85.3±1.1	83.2±2.0	93.3±1.7	92.9±1.4	73.0±1.6	65.8±1.9	84.2±2.1	84.8±1.7	82.9±0.9	75.3±1.9	84.5±1.2	83.7±1.4

**Table 5:** Ablation and optimization-strategy comparisons across three datasets. Results are reported as the mean ± standard deviation over five runs. The best results are underlined and the second are bold.

Variants	Chaoyang				Brain Tumor				Breast Tumor			
	ACC	Macro-F1	AUC-OVO	AUC-OVR	ACC	Macro-F1	AUC-OVO	AUC-OVR	ACC	Macro-F1	AUC-OVO	AUC-OVR
No SRA & No GCR	77.5±1.8	73.2±2.2	91.2±2.0	91.2±2.2	73.7±1.4	72.6±1.8	92.0±1.3	90.3±1.8	75.6±2.7	70.4±3.9	84.2±1.8	83.5±3.1
Teacher-Only	79.5±1.1	75.2±1.2	91.7±2.6	92.6±0.7	82.0±2.2	80.6±1.9	94.5±2.5	95.4±2.3	76.0±2.5	72.9±1.9	84.7±2.4	84.0±3.0
Label-Only	76.9±2.3	71.5±2.3	87.9±3.1	89.1±2.2	82.5±1.7	81.9±2.5	93.8±2.8	95.5±2.6	80.6±2.3	78.5±1.7	90.3±2.1	90.0±1.6
No-Prior	77.0±1.8	72.0±1.4	89.1±1.7	90.4±0.9	80.0±2.0	78.4±2.3	93.4±2.5	92.8±2.0	82.1±2.2	77.3±1.7	90.5±2.5	89.7±2.5
Fixed-Alpha (0.50)	77.7±1.7	72.5±2.7	91.5±3.0	93.0±2.2	77.7±1.6	75.3±1.7	91.5±1.2	91.5±2.6	78.7±1.7	76.4±1.8	89.1±2.5	88.9±1.2
MaxProb-Alpha	78.0±2.4	72.2±1.6	89.9±2.2	91.6±1.5	79.2±2.1	77.7±0.8	92.5±1.4	93.5±1.5	79.4±1.2	77.1±1.6	91.3±1.2	90.7±2.3
No GCR	77.2±2.6	72.6±1.6	90.3±2.4	91.2±1.9	82.2±1.0	81.6±0.5	94.3±0.8	94.5±1.3	77.5±1.8	74.1±1.9	92.5±1.6	92.0±1.5
Soft GCR	77.2±1.1	72.1±1.0	89.5±0.5	90.2±1.1	81.4±2.2	81.5±1.5	93.9±1.3	93.9±1.9	79.6±1.6	75.6±2.1	92.7±2.4	92.7±1.9
PCGrad	75.8±1.3	69.2±1.8	89.6±1.6	90.3±1.8	78.4±2.5	76.7±2.3	92.1±2.0	92.6±1.7	79.0±1.8	74.5±2.5	89.9±2.7	89.3±2.2
CAGrad	76.7±3.5	71.6±3.5	89.3±3.7	90.7±2.8	74.4±2.0	73.6±1.6	91.5±2.1	92.4±2.4	79.4±0.9	76.7±0.6	91.5±1.0	91.7±1.5
**GCR (δ=−0.2)**	77.2±1.3	72.0±1.3	89.4±1.6	91.5±1.5	79.8±1.2	77.8±1.9	92.8±1.6	93.7±1.1	74.3±2.6	70.7±1.9	83.9±2.3	85.0±1.9
GCR (δ=−0.1)	78.2±2.0	72.7±2.1	89.6±2.4	90.3±2.0	78.2±2.2	76.4±1.7	92.2±2.5	92.9±2.3	76.4±1.6	73.5±0.8	85.6±1.2	85.9±1.2
GCR (δ=0.1)	76.5±1.7	69.3±1.3	87.4±1.9	88.3±1.5	81.4±2.9	79.9±2.8	93.3±2.1	94.8±2.1	76.8±2.5	74.5±1.3	88.2±2.0	88.9±1.8
GCR (δ=0.2)	75.5±3.3	69.4±3.1	88.5±2.9	89.6±3.1	80.5±2.6	78.8±2.9	93.9±2.7	95.0±2.9	76.0±3.2	73.7±2.1	85.3±2.9	85.6±3.1
Baseline-2 (Expanded Training Set)	80.9±2.8	81.4±2.6	90.9±2.8	91.5±3.1	80.8±2.5	80.7±2.0	93.1±1.6	93.6±1.9	71.6±2.1	67.9±2.0	83.3±3.0	82.6±3.1
Vanilla KD (Standard Protocol)	80.8±1.2	76.8±1.1	91.4±1.5	92.1±1.7	80.9±1.2	79.7±1.2	91.3±1.3	91.5±1.5	80.2±2.9	78.6±2.4	91.2±2.5	90.4±2.5
SCOPE (Standard Protocol)	81.5±1.4	77.5±1.1	92.0±1.5	92.6±1.5	81.2±1.0	80.1±1.4	91.8±0.8	92.0±1.2	81.9±1.6	80.3±1.2	91.9±1.2	92.0±0.7
**SCOPE (δ=0)**	82.8±1.8	78.9±2.0	92.9±1.8	92.7±1.3	82.8±2.0	81.5±2.0	90.9±1.4	92.5±2.0	85.3±1.1	83.2±2.0	93.3±1.7	92.9±1.4

Note: SCOPE (Standard Protocol) uses the conventional KD setting in which teacher and student share the same training partition, whereas SCOPE (*δ* = 0) follows the proposed teacher-pretraining/student-training split used in the main experiments.

**Table 6:** Calibration, uncertainty, and robustness evaluation across three representative datasets.

Method	Chaoyang				Brain Tumor				Breast Tumor			
	ECE	ED-AUC	C-ACC	N-ACC	ECE	ED-AUC	C-ACC	N-ACC	ECE	ED-AUC	C-ACC	N-ACC
KD-FMV	0.084±0.007	78.1±1.6	77.7±1.2	72.9±1.8	0.071±0.006	80.6±1.8	80.0±2.0	74.6±2.2	0.096±0.009	75.4±2.1	79.2±1.9	72.8±2.4
SCOPE	0.052±0.005	84.2±1.3	82.8±1.8	79.4±1.6	0.046±0.004	86.1±1.4	82.8±2.0	79.9±1.9	0.061±0.006	82.7±1.7	85.3±1.1	82.1±1.5

Note: ED-AUC is the error-detection AUC (%) obtained using predictive entropy as the score. C-ACC and N-ACC denote clean and Gaussian-noise-perturbed ACC (%), respectively. Gaussian noise with *σ* = 0.05 is a controlled synthetic perturbation.

**Table 7:** Teacher classification accuracy across normalized teacher-entropy intervals on the test sets. Each entry reports the mean teacher accuracy ± standard deviation (%) over five runs.

Dataset	Normalized Teacher-Entropy Interval		
	[0.0, 0.33)	[0.33, 0.66)	[0.66, 1.0]
LC25000	98.48±1.40	92.37±2.06	77.15±3.82
HAM10000	90.25±2.38	78.59±1.86	53.87±4.31
Chaoyang	91.43±3.56	78.85±5.60	58.64±1.90
Multiple Myeloma	80.28±6.22	70.38±2.05	51.96±8.79
Brain Tumor	93.99±4.64	79.13±3.06	58.24±12.12
Kidney	95.85±1.64	87.77±2.73	77.82±10.13
Breast Tumor	92.81±3.32	78.96±3.12	60.36±7.99
Cataract	92.40±5.81	74.21±5.99	43.37±21.41
PAPILA	88.85±5.53	70.65±7.34	46.05±17.18

Note: Normalized teacher entropy is defined as $H(p_{i}^{t})/\log C$ and ranges from 0 to 1, with lower values indicating greater teacher confidence. Values are reported as the mean teacher accuracy ± standard deviation over five runs. Across all datasets, lower teacher-entropy intervals are associated with higher empirical teacher classification accuracy.

**Table 8:** GPU and CPU inference latency, model complexity, and classification performance on the LC25000 dataset. ACC is reported as the mean ± standard deviation over five matched runs. GPU latency was measured on an NVIDIA RTX A6000 GPU with 48 GB of memory, whereas CPU latency was measured on an Intel Core i7 processor. Entries marked with “–” indicate that the corresponding method could not be executed successfully in the CPU-only environment because of memory limitations, excessive latency, or unsupported operations. ↑: Indicates that larger values of this metric are better, ↓: Indicates that smaller values of this metric are better. The best results are underlined.

Method	GPU (s) ↓	CPU (s) ↓	Params (M) ↓	FLOPs (G) ↓	ACC (%) ↑
Baseline-1	0.033	0.061	11.17	9.42	78.9±2.3
Baseline-2	0.084	0.103	44.50	40.12	94.5±2.9
USKD	0.033	0.082	11.20	9.69	82.5±2.0
LSKD	0.034	0.078	11.19	9.79	97.1±1.3
OFAKD	0.033	0.085	11.21	9.88	83.2±1.8
UniDistill	0.033	0.082	11.20	9.74	93.2±1.0
SDKD	0.035	0.082	11.21	9.71	96.0±1.5
PETL	0.035	0.083	11.21	9.94	91.6±1.4
MCAD-KD	0.035	0.081	11.20	9.74	92.6±2.4
LDRLD	0.034	0.078	11.20	10.12	84.4±2.4
FoPro-KD	0.036	0.078	11.23	9.85	90.1±1.9
KD-FMV	0.035	0.078	11.19	9.89	91.0±2.1
AFA	0.048	–	98.03	102.69	84.2±1.3
DEPICT	0.059	–	84.94	102.27	92.2±1.2
ProtoPNets	0.086	–	97.06	94.06	91.1±1.7
Diff-Mix	0.061	–	90.12	75.73	84.7±1.3
SCOPE (Ours)	0.034	0.081	11.19	9.69	$\underline{98.3\pm 1.5}$

**Table 9:** Comprehensive performance comparison across various teacher–student configurations on nine medical image classification datasets. Results are reported as mean ± standard deviation over 5 matched runs. The best results are underlined.

**Teacher→Student**	**LC25000**				**HAM10000**				**Chaoyang**			
	**ACC**	**Macro-F1**	**AUC-OVO**	**AUC-OVR**	**ACC**	**Macro-F1**	**AUC-OVO**	**AUC-OVR**	**ACC**	**Macro-F1**	**AUC-OVO**	**AUC-OVR**
ResNet101→ResNet18	98.3 ± 1.5	98.7 ± 1.3	98.9 ± 1.3	98.5 ± 1.4	84.0 ± 2.3	71.0 ± 2.6	92.7 ± 2.1	95.1 ± 2.5	82.8 ± 1.8	78.9 ± 2.0	92.9 ± 1.8	92.7 ± 1.3
ViT-B→ResNet18	99.8 ± 0.3	99.9 ± 0.1	100.0 ± 0.0	100.0 ± 0.0	84.1 ± 0.8	73.2 ± 0.5	92.6 ± 1.2	95.5 ± 0.2	83.7 ± 0.7	80.1 ± 0.3	94.9 ± 0.5	95.8 ± 0.4
WideResNet101→ResNet18	99.7 ± 0.3	99.9 ± 0.1	100.0 ± 0.0	100.0 ± 0.0	84.9 ± 0.8	74.0 ± 0.9	92.3 ± 0.5	95.6 ± 0.2	81.7 ± 0.5	76.6 ± 1.6	93.9 ± 0.6	95.0 ± 0.7
ResNet101→ShuffleNetV2	99.8 ± 0.3	99.8 ± 0.0	100.0 ± 0.1	100.0 ± 0.0	82.1 ± 0.6	67.2 ± 0.6	88.6 ± 0.3	91.2 ± 0.3	81.6 ± 0.3	75.6 ± 0.8	89.7 ± 1.0	91.5 ± 1.3
**Teacher→Student**	**Multiple Myeloma**				**Brain Tumor**				**Kidney**			
	**ACC**	**Macro-F1**	**AUC-OVO**	**AUC-OVR**	**ACC**	**Macro-F1**	**AUC-OVO**	**AUC-OVR**	**ACC**	**Macro-F1**	**AUC-OVO**	**AUC-OVR**
ResNet101→ResNet18	64.1 ± 2.0	65.0 ± 2.4	82.4 ± 1.9	80.7 ± 1.2	82.8 ± 2.0	81.5 ± 2.0	90.9 ± 1.4	92.5 ± 2.0	98.5 ± 1.4	98.9 ± 1.3	98.8 ± 1.4	99.2 ± 1.3
ViT-B→ResNet18	62.6 ± 1.0	60.5 ± 0.6	80.2 ± 0.5	79.6 ± 0.4	83.2 ± 0.7	81.7 ± 0.4	91.4 ± 0.9	91.7 ± 0.4	99.3 ± 0.9	99.0 ± 0.3	100.0 ± 0.0	100.0 ± 0.0
WideResNet101→ResNet18	62.8 ± 1.0	60.5 ± 1.8	80.7 ± 0.4	79.9 ± 0.2	84.1 ± 0.5	82.7 ± 0.3	93.0 ± 0.8	93.3 ± 0.6	99.6 ± 0.4	99.4 ± 0.3	99.5 ± 0.1	99.6 ± 0.1
ResNet101→ShuffleNetV2	62.4 ± 0.8	61.7 ± 0.5	83.7 ± 0.6	83.0 ± 1.3	82.2 ± 0.7	81.1 ± 1.0	94.4 ± 1.0	94.8 ± 0.9	99.8 ± 0.3	99.7 ± 0.1	100.0 ± 0.0	100.0 ± 0.0
**Teacher→Student**	**Breast Tumor**				**Cataract**				**PAPILA**			
	**ACC**	**Macro-F1**	**AUC-OVO**	**AUC-OVR**	**ACC**	**Macro-F1**	**AUC-OVO**	**AUC-OVR**	**ACC**	**Macro-F1**	**AUC-OVO**	**AUC-OVR**
ResNet101→ResNet18	85.3 ± 1.1	83.2 ± 2.0	93.3 ± 1.7	92.9 ± 1.4	73.0 ± 1.6	65.8 ± 1.9	84.2 ± 2.1	84.8 ± 1.7	82.9 ± 0.9	75.3 ± 1.9	84.5 ± 1.2	83.7 ± 1.4
ViT-B→ResNet18	87.4 ± 1.1	86.1 ± 0.8	95.2 ± 0.4	94.8 ± 1.6	73.0 ± 1.0	64.5 ± 0.5	83.3 ± 0.8	83.2 ± 0.9	82.3 ± 1.1	73.4 ± 0.4	85.1 ± 0.7	86.0 ± 0.8
WideResNet101→ResNet18	85.3 ± 1.1	84.0 ± 0.8	95.2 ± 0.4	94.7 ± 0.8	72.2 ± 1.2	64.2 ± 1.1	85.4 ± 1.3	86.0 ± 1.2	82.6 ± 1.8	74.8 ± 0.4	81.0 ± 0.5	80.7 ± 0.7
ResNet101→ShuffleNetV2	85.5 ± 1.2	83.5 ± 0.9	92.8 ± 2.0	92.5 ± 1.8	70.5 ± 1.1	64.8 ± 0.3	81.7 ± 1.5	82.2 ± 0.7	81.9 ± 1.8	75.2 ± 0.9	85.9 ± 0.3	85.9 ± 0.4

**Table 10:** Sensitivity analysis of selected optimization hyperparameters on the Chaoyang validation set (%). Results are reported as the mean ± standard deviation over five runs. The best results are underlined.

Hyperparameter	Candidate Value	ACC	Macro-F1	AUC-OVO	AUC-OVR
β	0.2	80.8±1.0	77.1±1.3	91.9±1.1	91.5±1.3
	0.5	82.2±0.9	78.9±1.5	92.9±1.0	92.7±1.2
	0.8	82.1±1.8	77.9±2.0	92.8±1.6	92.0±1.3
	1.0	82.8±1.3	80.0±1.3	92.9±1.6	94.3±1.3
	1.5	80.9±1.1	78.3±0.9	93.0±1.3	92.2±1.6
	2.0	80.9±1.3	78.1±1.6	92.1±1.9	92.5±2.0
γ	0.2	80.3±0.7	76.7±1.1	91.0±1.3	91.5±0.9
	0.5	81.0±1.5	78.2±1.1	91.7±1.2	92.1±0.5
	0.8	83.2±1.2	80.0±1.8	93.3±1.6	92.7±1.5
	1.0	82.8±1.3	80.0±1.3	92.9±1.6	94.3±1.3
	1.5	82.1±0.8	77.7±0.8	92.9±1.5	94.0±1.4
	2.0	80.1±1.0	76.4±1.3	90.9±1.3	91.2±0.8
λ	0.2	81.9±1.3	77.8±0.8	93.0±1.1	93.3±0.7
	0.5	82.1±1.2	78.4±0.6	93.6±0.9	93.0±0.9
	0.8	82.8±1.3	80.0±1.3	92.9±1.6	94.3±1.3
	1.0	82.5±1.0	79.8±0.6	93.4±0.8	93.7±0.7
	1.5	80.9±1.0	78.2±1.2	91.8±0.8	91.7±0.8
	2.0	80.9±0.9	77.9±1.1	92.3±0.7	92.4±0.6
*T*	0.5	78.8±1.2	75.5±1.7	90.1±1.5	90.5±1.0
	1.0	80.8±1.9	77.4±1.9	92.4±2.0	92.7±1.5
	1.5	82.0±1.1	79.0±0.9	93.2±1.2	93.3±1.4
	2.0	82.8±1.3	80.0±1.3	92.9±1.6	94.3±1.3
	2.5	82.4±0.9	79.2±1.1	92.4±1.2	93.5±1.1
	3.0	81.3±1.5	78.3±1.2	91.2±1.1	92.0±1.0
	3.5	81.0±1.3	78.1±1.4	91.9±1.6	92.0±1.1
	4.0	79.5±1.2	76.1±0.6	90.7±1.2	90.7±1.2

Note: Each hyperparameter was varied individually while the remaining hyperparameters were fixed at the baseline values reported in Table 2. Results are reported as the mean ± standard deviation over five runs on the Chaoyang validation set.

## Data Availability

The publicly available datasets analyzed in this study can be accessed through their respective original repositories. The Multiple Myeloma dataset is not publicly available because it contains protected clinical information and is subject to institutional, ethical, and regulatory restrictions. The study data, associated code, and trained model weights may be made available by the corresponding author upon reasonable request and subject to appropriate institutional approval, in accordance with institutional policies, applicable NIH Data Management and Sharing requirements, and relevant regulatory and compliance requirements.
